# Assessment of Antibiotic Utilization Pattern in Treatment of Acute Diarrhoea Diseases in Bishoftu General Hospital, Oromia Ethiopia

**DOI:** 10.1155/2018/2376825

**Published:** 2018-05-02

**Authors:** Selamawit Tulu, Tarekegne Tadesse, Addisu Alemayehu Gube

**Affiliations:** ^1^Department of Pharmacy, College of Medicine and Health Sciences, Ambo University, P.O. Box 19, Ambo, Ethiopia; ^2^Department of Public Health, College of Medicine and Health Sciences, Arba Minch University, P.O. Box 21, Arba Minch, Ethiopia

## Abstract

**Background:**

Majority of acute diarrhoeal diseases are self-limiting and do not require routine treatment. Treatment with empirical antimicrobials is recommended only for dysenteric and invasive bacterial diarrhoea. Irrational use of antibiotics in treatment of acute diarrhoea is common in clinical practice worldwide. This study was carried out to assess the pattern of antibiotic use for acute diarrhoeal diseases in Bishoftu General Hospital, East Shewa Ethiopia.

**Methods and Materials:**

Institution based cross-sectional study was conducted from April 1 to April 30, 2016. Data were collected retrospectively from patients treated for diarrhoeal diseases from January 2015 to December 2015 using structured questionnaires and entered into SPSS (IBM 20) and descriptive statistics was carried out.

**Results:**

Among the 303 patients, 51.2% were males and 48.8% were females. Of them, 62% were children under five years. Two hundred sixty three (86.8%) patients received eight different types of antibiotics and cotrimoxazole (178 patients, 58.7%) was the most prescribed antibiotics, followed by ciprofloxacin (33, 10.9%) and amoxicillin (14, 4.6%). Based on the presence of blood in stools, 14.5% of cases were of invasive bacterial type. According to the recommendations of WHO, the rate of overuse of antibiotics was 72.3%.

**Conclusion:**

This study revealed that there was high overuse of antibiotics for both adults and children under five with acute diarrhoea in Bishoftu General Hospital. And Cotrimoxazole was the most prescribed antibiotic.

## 1. Introduction

An antibiotic is a drug that kills or slows the growth of bacteria [1]. Every week a new antibiotic started to be propelled into the market leaving no time for doctors to fully get familiar with new products while, at the same time, providing ample chances to microorganisms to develop different means of resistance to guarantee their survival [2]. Inappropriate antibiotic use is defined as a use that minimizes therapeutic impacts while it maximizes toxicity and the development of resistance. In Ethiopia, there are indications on the misuse of antibiotics by health-care providers, unskilled practitioners, and drug consumers. These coupled with rapid spread of resistant bacteria and inadequate surveillance contributed to the problem [3, 4].

Diarrhoea is defined as the passage of three or more loose or liquid stools per day (or more frequent passage than is normal for the individual). Frequent passing of formed stools is not diarrhoea, nor is the passing of loose, “pasty” stools by breastfed babies. There are three clinical types of diarrhoea: acute watery diarrhoea that lasts several hours or days, and includes cholera, acute bloody diarrhoea also called dysentery, and persistent diarrhoea which lasts 14 days or longer [5]. In both industrialized and developing countries, viruses are the predominant cause of acute diarrhoea, particularly in the winter season. Supportive rehydration therapy, associated with adequate nutritional support, is the cornerstone of therapy, regardless of the etiology and the severity of the process, and its prompt and early adoption is associated with a favorable outcome. Empirical antibiotic treatment is recommended since over 90% of cases of diarrhoea pathogens cannot be identified. But the clinical benefit of an empiric antibiotic treatment should be evaluated taking into account the risk of adverse event reactions and the risk of harmful eradication of normal flora [6].

Increased antibiotic use in the hospital is often associated with increased frequency of resistance. The antibiotic usage patterns exert a significant influence over the rates of resistance observed in multidrug-resistant nosocomial pathogens [7–10]. The increased resistance has increased the morbidity and mortality in many patients, and it has also increased the cost of treatment [3, 8]. Increased cost of health care will definitely jeopardize the capacity of the poor population to seek modern health care. Moreover, the proportion of inappropriate antimicrobial use in the hospital was more than 30% [10].

It is estimated that about 20% to 50% of all antibiotics use is inappropriate, resulting in increase of side effect, high cost, and high rate of antimicrobial resistance (AMR) in community pathogens [11]. The most common inappropriate antibiotic use is for viral and self-limiting infection in the acute diarrhoea. Viral pathogens such as rotavirus account for 70% to 80% of all diarrhoeal episodes [12]. In majority of acute diarrhoea, the cause usually remains unknown because of self-limiting nature of disease and difficulty and delay in identifying pathogen, so routine use of antimicrobial is not recommended [13]. The joint statement by WHO and United Nation International Children Emergency Fund (UNICEF) in 2004 recommended the use of low osmolality ORS along with zinc for treatment of acute diarrhoea in children [14]. Antibiotics are recommended only for acute bloody diarrhoea or dysentery. Unfortunately, reports from different parts of the world reported that misuse of antibiotic are common in treatment of diarrhoea [15]. Understanding the extent and pattern of antimicrobial use for acute diarrhoea in community is important for defining regional intervention program to promote rational use of antimicrobials and then limit the spread of AMR and reduce the cost of therapy for acute diarrhoea. Hence, this study was conducted in Bishoftu General Hospital to obtain information on antibiotic utilization pattern for treatment of acute diarrhoeal diseases.

## 2. Materials and Methods

### 2.1. The Study Setting and Period

Bishoftu is a town and separate woreda of Ethiopia, lying southeast of Addis Ababa. It was formerly known as Debre Zeyit; however, since the late 1990s, it has been officially known by the Oromo name Bishoftu, which was its name until 1955. The town is located in the Misraq Shewa Zone of the Oromia Region. It is located 47.9 kilometres (29.8 mi) southeast of Addis Ababa along its route 4 highway. It lies at 9°N latitude and 40°E longitude at an altitude of 1950 m above sea level. The average maximum and minimum temperature of the area is 34.7°C and 8.5°C, respectively, and average relative humidity is 61.3%. The rainfall is bimodal. It receives an annual rainfall of 1151.6 mm of which 84% is received during the long rainy season covering June to September and the remaining in the short rainy season extending from March to May. Bishoftu General Hospital was established in 1941 GC and at that time, it was serving as prison camp for the Italian military. Now the hospital is giving health service for people from three towns and five districts, and for the total of 1.2 million people and it has 102 beds distributed in different wards, such as medical, surgical, gynecology, and emergency department, and its catchments area is around 3,600 square kilo meters. The hospital also provides different services like, OPD service, MCH service, psychiatric clinic, and medical follow-up or chronic illness clinics. It has a total of 174 health professionals. The hospital logs roughly 154,000 visits a year with 4000 annual admissions. The study was carried out from April 1 to April 30, 2016.

### 2.2. Study Design

Institution-based cross sectional study was conducted, and data were collected retrospectively.

### 2.3. Source Population

All diarrhoeal patient records in Bishoftu General Hospital were the source.

### 2.4. Study Population

Diarrhoeal patient charts that are diagnosed and treated for acute diarrhoeal disease in BGH from January to December 2015 were used.

### 2.5. Sample Size Determination and Sampling Technique

The sample size for this study was determined using single population proportion formula, assuming, 95% confidence interval and 5% margin of error and a prevalence of 50% to get possible minimum large sample size. So, a total of 384 was calculated, and since the number of population was less than 10,000 and finite which is 1436 acute diarrhoeal disease patient charts, adjusting the population size to 1436 and calculating the sample size in STAT CALC of EPI Info software gives the final sample size of *303* acute diarrhoeal disease patient charts.

To identify the patient charts, a systematic sampling technique was used. First, every patient chart numbers diagnosed with acute diarrhoea disease documented in OPD registration book was counted and recorded; then sampling interval was determined by dividing the total number of patient charts by sample size which gives the interval *k* = 5 and every 5th chart was selected. The first patient chart was selected using lottery method from first up to 5th patient chart starting from the time order of the records.

### 2.6. Data Collection Tools and Procedures

The data were collected using check list which is structured based on the study objective and designed as simple as possible. Data collection was done using patient chart, laboratory result, and prescriptions on the chart and prescriber profile.

### 2.7. Data Processing and Analysis

After checking for completeness and consistency, data were entered into SPSS (IBM 20), and descriptive statistics was carried. And data were presented using narratives, tables, and figure.

## 3. Results

### 3.1. Sociodemographic Characteristics of the Patients

There were 1436 patient records diagnosed with acute diarrhoeal diseases within study periods (from January 2015 to December 2015). A total of 303 patient records were reviewed. Among 303 patients, 155 (51.2%) were males and 148 (48.8%) were females. Children under five years of age were 62% and older were 5% (Table 1).

### 3.2. Clinical Characteristics

**Figure 1:**
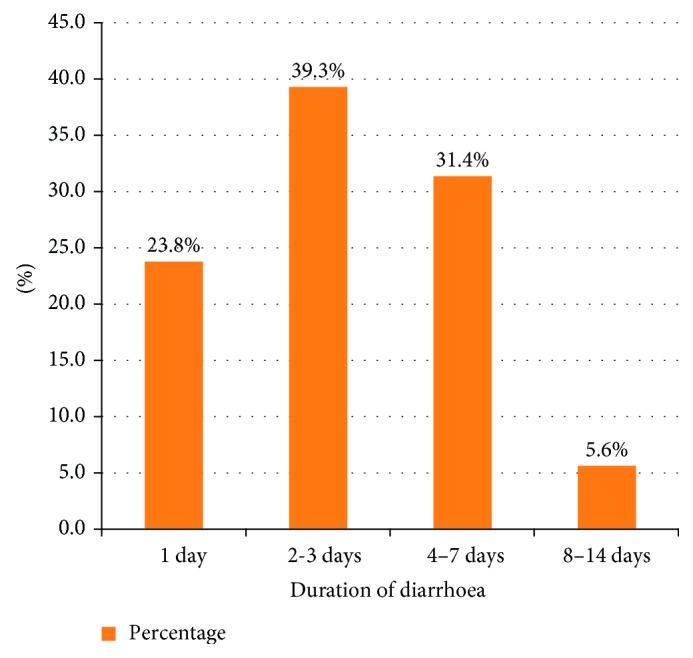
Duration of diarrhoea from onset to treatment for patients diagnosed with acute diarrhoea at Bishoftu General Hospital from January 2015 to December 2015.

A review of history of the cases shows that 119 (39.3%) patients had experienced the illness for 2 to 3 days (Figure 1). Most of the patients had reported to having sickness associated with their diarrhoea, such as fever 133 (43.9%), vomiting 185 (61.1%), cough 18 (6%), chills 3 (1%), headache 15 (5%), abdominal cramp 94 (31.1%), and loss of appetite 14 (4.7%). Concerning the degree of dehydration, 77 (25.4%) patients had mild to moderate dehydration. Only 2 (0.7%) had severe dehydration, which required intravenous fluid therapy (IV).

### 3.3. Stool Characteristics

**Figure 2:**
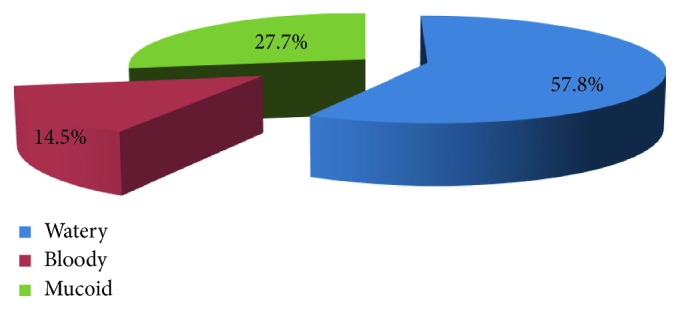
Stool characteristics for acute diarrhoea patients at Bishoftu General Hospital, Bishoftu, from January 2015 to December 2015.

Two hundred fourteen (70.6%) patients had a stool examination ordered. 103 (48.1%) of the 214 stool specimens were positive; of these, 68 (31.8%) were with unspecified bacteria and 35 (16.4%) contain amoeba, giardia, and ascariasis. Out of 303 patients, 85.5% of stools were nonbloody and 14.5% have blood in stools (Figure 2).

### 3.4. Treatment Patterns of Acute Diarrhoeal Diseases

As patients' records show, the number of antibiotics prescribed for single patient ranged from 0 to 3 drugs. About 86.8% of cases received at least one antibiotic drug, while 13.2% of them received no antibiotics, 78.8% received one, 7.3% received 2, and 0.7% received 3 antibiotics during the episode of diarrhoea.

Eight types of antimicrobials were prescribed. Cotrimoxazole was the most commonly prescribed drug (58.7%), followed by ciprofloxacillin (10.9%), amoxicillin (4.6%) and metronidazole (2%) (Table 2).

In the same manner, of 188 under five children, 174 (92.6%) were prescribed with at least one antibiotic (Table 3).

Of 303 patients, 134 (44.2%) patients were prescribed with ORS while 2 patients prescribed with IV fluid for treatment of dehydration. Other medications prescribed for these patients were paracetamol 102 (33.7%), Albendazole 12 (4%), Mebendazole 9 (3%), Ibuprofen 6 (2%), Diclofenac 6 (2%), Multivitamin 6 (2%), Tramadol 6 (2%), metoclopramide 3 (1%), Omeprazole 3 (1%), Tindazole 3 (1%), and Hyosine 3 (1%).

### 3.5. Adherence to Standard Treatment Guidelines

Seen against treatment guidelines, 84 (27.7%) cases were appropriately treated while 219 (72.3%) cases were inappropriately treated (Table 4).

### 3.6. Prescriber Profile on Acute Diarrhoea

Mostly, physicians treated acute diarrhoeal patients. Around 100 (33%) patient records had no name of prescribers. The proportion of appropriate antimicrobial use among general practitioners was higher than that of nurses (Table 5).

## 4. Discussion

This institution-based cross sectional study has investigated the pattern of antibiotic use for acute diarrhoeal diseases in Bishoftu General Hospital, East Shewa, Ethiopia.

In this study, of the patients treated for acute diarrhoea, 86.8% of patients have received at least one antibiotic drug. This find is far higher than the findings of the studies carried out in Ujjain, India, China, and Thailand where 71%, 60.8%, and 45.1% received antibiotic drug for acute diarrhoeal disease, respectively [16–18]. This might be because health professionals in India, China, and Thailand may have a good knowledge and follow the standard treatment guideline than health professionals in Bishoftu General Hospital. Because India, China, and Thailand have relatively a good health-care setting than Ethiopia.

Similarly, in this study, 92.6% of children under five years with acute diarrhoea have received at least one antibiotic drug. This finding is higher than the findings of the studies conducted in Central Region Province of Thailand, Delhi, India, and Puducherry, India, where the percentage of patients prescribed on antibiotics were 72.6%, 64%, and 22%, respectively [19–21]. This might be attributable to the fact that health professionals in Bishoftu General Hopsital may be tempted by the expectation of the children's parents than parents in mentioned countries, because the communities in Thailand and India are relatively more developed in every aspect than communities in Ethiopia particularly in Bishoftu and the surroundings.

In the current study, the percentage of acute diarrhoeal patients treated inappropriately when seen against standard treatment guideline was 72.3%. This finding is far higher than the finding of the study carried out in China and Thailand where 51.3% and 48.9% of patients, respectively, treated inappropriately [17, 18]. This might be because the health-care system is more advanced in China and Thailand than in Bishoftu. In contrast to our study, the other study conducted in Thailand, has indicted that 73.8% of appropriate antibiotic use for diarrhoeal disease treatment [22].

In this study, cotrimoxazole is the most commonly prescribed drug (58.7%). This finding is in line with the finding of the study conducted in Central Region Province, Thailand, where in 51% of cases cotrimoxazole was the most commonly prescribed drug [19].

## 5. Conclusion

This study revealed that there was high overuse of antibiotics for both adults and children under five years with acute diarrhoea in Bishoftu General Hospital. And cotrimoxazole was the most prescribed antibiotic.

## Figures and Tables

**Table 1:** Sociodemographic characteristics of acute diarrhoea patients at Bishoftu General Hospital, Bishoftu town from January 2015 to December 2015.

Variables	Frequency	Percentage
*Sex*		
Male	155	51
Female	148	49
*Age*		
Less than 5 year	188	62
5–12 years	48	16
13–40 years	30	10
41–65 years	22	7
>65 years	15	5

**Table 2:** Antibiotics prescribed for acute diarrhoea patients at Bishoftu General Hospital, Bishoftu town, from January 2015 to December 2015.

Variables		Frequency	Percentage
Class of antibiotics	Antibiotics		
Floroqunilones	Ciprofloxacillin	33	11
Pencillins	Amoxicillin	14	5
Tetracycline	Doxycycline	1	0.3
Aminoglycoside	Gentamycin	1	0.3
Cephalosporins	Ceftriaxone	3	1
	Cephalexin	3	1
Others	Cotrimoxazole	178	59
	Metronidazole	6	2
Combinations	Metronidazole + Cotrimoxazole	3	1
	Metronidazole + Ciprofloxacillin	8	3
	Cotrimoxazole + Amoxacillin	6	2
	Ciprofloxacillin + Doxycycline	5	2
	Metronidazole + Ciprofloxacillin + Cotrimoxazole	2	1
Total		263	87

**Table 3:** Antibiotics prescription by age groups and stool characteristics for acute diarrhoea patients at Bishoftu General Hospital, Bishoftu town, from January 2015 to December 2015.

Variables					Frequency
Antibiotics prescribed	Age groups	Stool			
		Watery	Bloody	Mucoid	Total
Yes	<5	108	25	41	174
	5–12	13	7	8	28
	13–40	17	6	4	27
	41–65	14	1	4	19
	>65	10	5	0	15
	Total	162	44	57	263

No	<5	10		4	14
	5–12	1		19	20
	13–40	1		2	3
	41–65	1		2	3
	Total	13	0	27	40

*Note*. Mixed watery and mucoid diarrhoea is grouped under mucoid.

**Table 4:** Antibiotic utilization patterns based on treatment guidelines for acute diarrhoeal diseases in Bishoftu General Hospital Bishoftu town, January 2015–December 2015.

Antimicrobial usage	Frequency	Percentage
*Appropriate*	84	28
(i) Given antimicrobial for bloody diarrhoea	44	15
(ii) Given Iv fluid forsevere diarrhoea	2	1
(iii) Not given antimicrobialfor nonbloody diarrhoea	38	13

*Inappropriate*	219	72
(i) Not given antimicrobialfor bloody diarrhoea	0	0
(ii) Given antimicrobial for nonbloody diarrhoea	219	72

**Table 5:** Antibiotic usage for acute diarrhoeal diseases of health professionals in Bishoftu General Hospital, Bishoftu town, January 2015–December 2015.

Profession of prescriber	Prescriptions		Antibiotic usage			
	Number	%	Appropriate		Inappropriate	
			Number	%	Number	%
Physician (general practitioners)	134	44	49	37	85	63
Nurse (with BSc. degree)	69	23	14	20	55	80
Unknown	100	33	21	21	79	79
Total	303	100	84		219	
